# Analysis and Characterization of *Staphylococcus aureus* Small Colony Variants Isolated From Cystic Fibrosis Patients in Austria

**DOI:** 10.1007/s00284-016-0994-z

**Published:** 2016-01-28

**Authors:** Lilian Masoud-Landgraf, Gernot Zarfel, Tanja Kaschnigg, Simone Friedl, Gebhard Feierl, Ute Wagner-Eibel, Ernst Eber, Andrea J. Grisold, Clemens Kittinger

**Affiliations:** Institute of Hygiene, Microbiology and Environmental Medicine, Medical University Graz, Neue Stiftingtalstraße 2, 8010 Graz, Austria; Department of Paediatrics and Adolescent Medicine, Medical University of Graz, Graz, Austria

## Abstract

Cystic fibrosis (CF) is the most common hereditary lung disease in the Caucasian population, characterized by viscous bronchial secretion, consecutive defective mucociliary clearance, and unavoidable colonization with microorganisms. Besides *Pseudomonasaeruginosa*, *Staphylococcusaureus* is the most common bacterial species colonizing the CF respiratory tract. Under antibiotic pressure *S.aureus* is able to switch to small colony variants (SCV). These small colony variants can invade epithelial cells, overcome antibiotic therapy inside the cells and can be the starting point for extracellular recolonization. The aim of the present study was the isolation and characterization of *S. aureus* small colony variants from Austrian cystic fibrosis patients. Samples collected from 147 patients were screened for the presence of *S. aureus* wild-type and small colony variants. Antibiotic susceptibility testing and determination of the small colony variants causing auxotrophism were performed. Wild-type isolates were assigned to corresponding small colony variants with spa typing. In total, 17 different small colony variant isolates and 12 corresponding wild-type isolates were obtained. 13 isolates were determined thymidine auxotroph, 2 isolates were auxotroph for hemin, and none of the tested isolates was auxotroph for both, respectively. The presence of SCVs is directly related to a poor clinical outcome, therefore a monitoring of SCV prevalence is recommended. This study revealed rather low SCV ratios in CF patients compared to other countries.

## Introduction

Cystic fibrosis (CF) is the most common hereditary lung disease in the white (Caucasian) population [1, 2, 13, 19, 30]. CF is caused by mutations in the gene that encodes the cystic fibrosis transmembrane conductance regulator (CFTR) protein [9, 23]. Abnormal CF transmembrane conductance regulator function initiates a pathophysiologic cascade of chronic airway inflammation and suppurative infection resulting in progressive pulmonary insufficiency. Ultimately, 95 % of patients with CF succumb to respiratory failure.

In the last years, studies revealed an association between *Pseudomonas aeruginosa(P. aeruginosa)* and CF lung function decline [12, 18], so the microbial researches and therapy strategies focused on *P. aeruginosa*. However, recent studies describe an increase of *Staphylococcus aureus* (*S. aureus)* prevalence in CF, both methicillin-susceptible and methicillin-resistant (MRSA) strains [14]. Latest studies on children with CF noted similar inflammation and lung function decline during infection with *S. aureus* compared with *P. aeruginosa* [15, 28, 42].

*S*. *aureus* is thought to persist in CF patients over years and adapts to this environment. One occurrence of adaption is the so-called small colony variant (SCV) phenotype. This phenotype has been reported from various bacterial and fungal species and the transformation to a SCV phenotype is triggered by several stress factors [8, 20]. The genetic background of the SCV phenotypes is based on mutations, that lead to auxotrophy in metabolic pathways. Three major types of *S. aureus* SCVs have been found in CF isolates, namely strains that are auxotroph for thymidine, menadione, or hemin [10, 41].

The switching to the SCV phenotype enables *S. aureus* to hide inside host cells and to escape from immune responses. The reduced doubling speed and alteration of pathways of SCVs also lead to an increased antibiotic resistance [3, 16, 44]. The intracellular activity of SCV phenotypes is additionally characterized by a lack of typical *S. aureus* pathogenic functions. The only persistence of SCVs does not seem to be a problem for the patient, but the instability of most SCVs bears the risk of reconversion to the fast growing and virulent wild type [25, 39]. So SCV strains have significant clinical importance and in some cases they can even persist for up to 50 years in the patient [31].

*S. aureus* SCVs are difficult to detect, as they generally grow slowly and often need several days to become visible on agar plates. They have altered drug-resistance profiles, such as an increased resistance to aminoglycosides [33, 36]. Currently, most clinical laboratories are unable to detect *S.aureus* SCVs, as adapted culture methods are not implemented in routine diagnostics [3, 44]. Results of susceptibility testing by disk diffusion or automated methods are often invalid or even not possible. The colonies are too small to be seen on agar plates or to be detected by automated systems [24].

Patients at risk of SCV *S. aureus* infection are patients with long-term antibiotic therapy [29], so besides from patients with CF also patients with endocarditis, pneumonia, soft tissue infections, osteomyelitis, and severe bacteraemia. The frequency of SCV recovery from clinical specimens ranges from 1 to 30 % in CF patients [33].

The aim of the present study was a first epidemiological look for SCVs in the airways of CF patients in Southeast Austria. All isolates were characterized by *spa* typing. Additionally their antibiotic susceptibility and the underlying auxotrophism were tested, in order to provide a characterization of CF SCV *S. aureus* strains in our region.

## Materials and Methods

Clinical isolates were obtained at the CF center at the Respiratory and Allergic Disease Division, Department of Paediatrics and Adolescent Medicine (Medical University of Graz), between January 2011 and December 2013. All CF patients of the center in Graz (ambulant and stationary patients, including all CF patients under treatment in Southeast Austria) are microbiologically screened at least four times per year. All *S. aureus* isolates included in this study were investigated at the CF Laboratory at the Institute of Hygiene, Microbiology and Environmental Medicine (IHMEM, Medical University of Graz).

### Collection of Bacterial Isolates

Sputum samples and mucoid BAL fluid samples were immediately processed with Sputasol^®^ (Oxoid) containing the sputum liquefying agent dithiothreitol in a weight ratio of 1:2, incubated at room temperature for 10–15 min and vortexed. This was followed by a serial dilution up to a 10^5^ dilution. 100 µl of the liquefied and diluted suspensions were plated onto TSA (Tryptocase-Soja-Agar, bioMérieux, Austria), Mac Conkey-Agar (bioMérieux, Austria), and SAID (*Staphylococcus aueus* identification-Agar, bioMérieux, Austria). 50 µl of the liquefied origin were plated on Chocolate-Agar (bioMérieux, Austria), BCSA-Agar (bioMérieux, Austria), Schaedler-Agar (bioMérieux, Austria), and Meropenem and Polymyxin-Agar (IHMEM, Medical University Graz). All agar plates were incubated at 35 °C for 72–120 h and up to 9 days at room temperature. The first screening for SCV *S. aureus* was carried out after 72 h. On TSA and blood-agar, non-hemolytic, non-pigmented, small and fried egg colonies were suspected to be *S. aureus* SCVs, on SAID all colonies were suspicious to be *S. aureus* SCVs. All conspicuous colonies were inoculated on blood and Schaedler-Agar [21]. If the colonies were normal-sized hemolytic and pigmented on Schaedler-Agar and not on blood-agar, they were considered to be *S. aureus* SCVs. These isolates were tested for species identification by MALDI-TOF MS Axima™ Assurance (Shimadzu, Japan). For this purpose, one colony of each isolate was directly spotted on the FlexiMass MALDI target plate. For automatic measurement and identification the SARAMIS™ (spectral archived microbial identification system) database application from bioMérieux was applied. Isolation of all other bacterial species was done as described previously [26].

### *Spa* Typing

*Spa* typing is based on sequencing the polymorphic X-region of the protein A gene (*spa*). This is a well-established technique and the results can be compared with databases. The determined *spa* types can be assigned to distinct groups with known origin and characteristics [10].

### Detection of *mec*A Gene

To confirm MRSA genotype, the presence of *mec*A was analyzed in all isolates with the BD MAX™ System (Beckton Dickinson Austria, Vienna), using the “BD MAX MRSA assay” according to the manufacturer’s instructions.

### Auxotrophism Test

SCV auxotrophism was tested using agar disk diffusion test according to Maduka-Ezeh et al. on Mueller–Hinton agar for thymidine, hemin, and menadione [37]. To identify potentially double auxotrophic strains, the agar plates were also loaded with all three test substrates additionally [4, 32, 35, 38].

### Determination of Antibiotic Resistance

Antibiotics tested were penicillin, oxacillin, gentamicin, erythromycin, clindamycin, ciprofloxacin, tetracycline, rifampicin, fusidic acid, vancomycin, linezolid, and mupirocin. Resistance testing was performed as recommended by the European Committee on Antimicrobial Susceptibility Testing (EUCAST, http://www.eucast.org/clinical_breakpoints/).

For the normal *S. aureus* strains, determination of the minimal inhibitory concentration (MIC) was performed by using VITEK-2 (bioMérieux, Solna, Sweden) and disk diffusion method, and by E-test (bioMérieux, Solna, Sweden). For the determination of the antimicrobial susceptibilities of the SCV *S. aureus* strains resistance was tested on brain–heart infusion (BHI) agar [21].

## Results

### Collection of Bacterial Isolates

Samples originated from 147 patients (75 male, 72 female). The patients’ age ranged from 10 months to 56 years, with a median age of 19 years. Samples included sputa (*n* = 1452), nasal swabs (*n* = 518), throat swabs (*n* = 466), and bronchoalveolar lavage (BAL) fluids (n = 58). In total, 1399 *S. aureus* isolates could be recovered in 127 patients (86.4 %), two of them were tested phenotypically and genotypically (*mec*A gene) positive for MRSA. 12 patients harbored SCV *S. aureus* isolates including the two MRSA positive patients. So, 8.2 % of the CF patients and 9.4 % of *S. aureus* positive CF patients were positive for *S. aureus* SCVs, respectively. 74 *S. aureus* (27 SCV and 47 wild types) could be isolated from the 12 SCV positive patients during the study period.

Only eight (66.7 %) of the patients with SCV strains were co-colonized with *P. aeruginosa*, whereas 95 of 127 (74.8 %) of *S*. *aureus* positive patients were also positive for *P. aeruginosa.* None of them was co-colonized with *Haemophilusinfluenzae* and three (18.8 %) of them were positive for *Stenotrophomonasmaltophilia*.

### *Spa* Typing

In total, 21 different *spa* types could be detected. SCV *S. aureus* revealed 13 *spa* types; 10 of these *spa* types could also be identified in wild-type *S. aureus* isolates from the same patient (Table 1). Three SCV *spa* types could not be assigned to a wild-type strain and 8 *spa* types were only present in wild type (data not shown). Only two *spa* types (t002 and t085) were present in more than one patient (Table 1).

**Table 1:** Antibiotic resistance pattern, type of auxotrophism, and spa types of the SCV isolates of 12 patients

SCV	Pat Nr	SCV isolation date	Auxotrophism	*Spa*-type	Resistance	Isolation date of the paired *S. aureus* strain
1	1	28.02.2013	Thymidine	t12308	P, AM, E, CC, GM	28.05.2010 15.12.2010 05.11.2013
2	2	07.02.2013	Thymidine	t209	P, AM, E, CC	28.08.2010 07.12.2011 29.06.2012
3	3	25.02.2013	Thymidine	t012	P, AM	Paired strain could not be found
4	4	17.10.2013	Thymidine	t732	E, CC	04.10.2010
5	5	07.11.2013	Thymidine	t002	OXA, P, AM, E, CC, GM, NN	28.11.2012 07.11.2013
6	5	30.08.2012	Thymidine	t355	OXA, P, AM, GM, RIF	14.04.2010 07.07.2010 02.11.2010 21.02.2011
7	6	08.07.2013	Thymidine	t8012	P, AM, E, CC	20.07.2010 28.10.2013
8	7	11.09.2012	Thymidine	t004	GM	25.07.2011 30.07.2013
9	8	11.04.2013	Thymidine	t085	P, AM	Paired strain could not be found
10	9	08.08.2012	Thymidine	t085	P, AM	09.05.2012
11	9	16.03.2013	Not known	t085		
12	9	15.05.2013	Hemin	t085	P, AM, GM	
13	9	20.09.2011	Thymidine	t645	P, AM, E, CC, GM	Paired strain could not be found
14	9	13.02.2013	Thymidine	t015	P, AM, CIP	25.05.2010 25.01.2011
15	10	25.06.2013	Not known	t2249	P, AM, E, CC, GM	02.11.2010 17.10.2012 16.07.2013
16	11	05.09.2013	Hemin	t002	P, AM, E, CC	10.08.2010
17	12	12.09.2012	Thymidine	t9847	P, AM, E, CC	12.07.2010 06.11.2012 20.11.2013

*OXA* oxacillin, *P* penicillin, *AM* ampicillin, *E* erythromycin, *CC* clindamycin, *GM* gentamicin, *NN* tobramycin, *CIP* ciprofloxacin, *RIF* rifampicin

*S. aureus* SCV were classified as identical if they had the same *spa* type and the same auxotrophism. 10 out of 12 CF patients (patients 1, 2, 3, 4, 6, 7, 8, 10, 11, and 12) harbored one SCV isolate. From two patients (patients 5 and 9) SCV *S. aureus* with different wild-type strains could be isolated. The isolated strain of patient 9 was the only one that had developed different SCV phenotypes (Table 1).

### Auxotrophism

Thirteen of the tested 17 isolates showed thymidine auxotrophism, and two times hemin dependence was recovered; menadione auxotrophism could not be linked to any isolate. Two isolates showed no response to the substrates and the auxotrophism could therefore not be addressed (Table 1). In contrast to the others, the t085 isolates of patient 9 showed SCV phenotype differentiation into thymidine, hemin, and a non-assignable type of auxotrophism. This *spa* type was also the only one that had developed different SCV phenotypes.

### Antibiotic Susceptibility of SCVs

Antibiotic susceptibility was determined for 17 strains. The highest resistance rate was observed for penicillin/ampicillin with 14 isolates (82.4 %). Resistance against erythromycin/clindamycin was present in nine isolates (52.9 %). On the basis of MIC testing with E-test, two isolates (11.7 %) were oxacillin resistant and therefore MRSA. All tested isolates were susceptible to tetracyclin, fusidic acid, linezolid, vancomycin, and mupirocin (Table 2).

**Table 2:** Antibiotic resistance within the SCV isolates

Antibiotic	Nr. of S	%S	Nr. of R	%R
Ampicillin	3	17.6	14	82.4
Penicillin	3	17.6	14	82.4
Oxacillin	15	88.3	2	11.7
Amoxicillin/clavulanic acid	15	88,3	2	11.7
Piperacillin/tazobactam	15	88.3	2	11.7
Gentamicin	10	58.8	7	41,2
Tetracyclin	17	100	0	0
Ciprofloxacin	16	94.1	1	5.9
Erythromycin	8	47.1	9	52.9
Clindamycin	8	47.1	9	52.9
Fusidic acid	17	100	0	0
Rifampicin	16	94.1	0	5.9
Linezolid	17	100	0	0
Vamcomycin	17	100	0	0
Mupirocin	17	100	0	0

*Nr.S* number of sensitive isolates,  %*S* percentage of sensitive isolates, *Nr* of *R* number of resistant isolates,  %*R* percentage of resistant isolates

## Discussion

Over a period of long-term infection, *S. aureus* may undergo extensive adaptation to the lung environment in CF patients [17]. One mentionable effect of this adaptation is the transition toward a slow-growing low virulence state, the small colony variant. SCVs mutants enable advantages under chronic infection conditions. For example, SCVs have the ability to survive intracellularly, where they are protected against phagocytosis and from extracellular antimicrobial agents. So, they overcome different environmental pressures (antibiotic treatment or bacterial competitors). The relation between the presence of SCVs and a poor clinical outcome has already been reported in several studies, with patients harboring SCV variants having lower blood oxygen levels and a significantly lower FEV1 score [5, 34].

Long-term treatment with trimethoprim/sulfamethoxazole (SXT) or aminoglycosides favors the emergence of thymidine-dependent SCVs in CF patients [7, 43]. This is in concordance with our data, as the patients in our study had previously been treated with SXT.

The prevalence of thymidine-dependent SCV strains in CF patients has already been described [6, 11, 22]. We also found a majority of thymidine-dependent strains, whereas not a single menadione-dependent strain could be detected. The reason for the absence of menadione auxotroph SCVs might be the extremely slow growth of the menadione auxotroph SCVs which could have been overgrown by faster growing variants.

Recent publications e.g., Melter et al. reported that SCV variants are much more resistant to antibiotics than the non-SCV *S. aureus* isolates [29]. In contrast, we could not find any difference in the resistance rates between wild-type *S. aureus* strains and SCV *S. aureus* strains.

Twelve patients were SCV positive, so the SCV *S. aureus* prevalence was 8.1 %, lower than previously reported from CF studies in other countries [45]. The prevalence of MRSA also is very low in our center. Oxacillin resistance was found in 11.7 % of the SCVs. This could be related to local infection control and the low incidence of nosocomial *S. aureus* acquisition in our CF center; multiple antibiotic-resistant *S. aureus* SCVs was not a common occurrence in this study [27, 43].

The suggested inductive effect on *S. aureus* SCV via *P. aeruginosa* co-infection could not be verified in our study but this might be related to the relatively low number of *S. aureus* SCV isolates [40, 43].

In conclusion, this first report on *S. aureus* SCVs from an Austrian CF center revealed a high number of *S. aureus* positive CF patients but rather low SCV prevalence. A tight cooperation between clinicians and microbiological laboratory is essential for a successful management of the underestimated SCV challenge.
